# Clinical, metabolic, and molecular genetic characterization of hereditary methemoglobinemia caused by cytochrome b_5_ reductase deficiency in 30 dogs

**DOI:** 10.1038/s41598-020-78391-2

**Published:** 2020-12-08

**Authors:** J. A. Jaffey, N. S. Reading, O. Abdulmalik, R. Kreisler, G. Bullock, A. Wiest, N. A. Villani, T. Mhlanga-Mutangadura, G. S. Johnson, L. A. Cohn, N. Isaza, J. W. Harvey, U. Giger

**Affiliations:** 1 Department of Specialty Medicine, College of Veterinary Medicine, Midwestern University, Glendale, AZ USA; 2 Department of Veterinary Medicine and Surgery, Veterinary Health Center, University of Missouri, Columbia, MO USA; 3 Institute for Clinical and Experimental Pathology, ARUP Laboratories, Salt Lake City, UT USA; 4 Division of Hematology, The Children’s Hospital of Philadelphia, Philadelphia, PA USA; 5 Department of Pathology and Population Medicine, College of Veterinary Medicine, Midwestern University, Glendale, AZ USA; 6 Department of Veterinary Pathobiology, College of Veterinary Medicine, University of Missouri, Columbia, MO USA; 7 Section of Medical Genetics (PennGen), School of Veterinary Medicine, University of Pennsylvania, Philadelphia, PA USA; 8 Department of Small Animal Clinical Sciences, College of Veterinary Medicine, University of Florida, Gainesville, FL USA; 9 Department of Physiological Sciences, College of Veterinary Medicine, University of Florida, Gainesville, FL USA

**Keywords:** Biochemistry, Genetics, Molecular biology, Diseases, Medical research, Molecular medicine

## Abstract

Genotype–phenotype correlations of humans and dogs with hereditary methemoglobinemia are not yet well characterized. We determined total hemoglobin and methemoglobin (MetHb) concentrations, cytochrome b_5_ reductase (CYB5R) enzyme activities, genotypes, and clinical signs in 30 dogs with persistent cyanosis without cardiopulmonary disease. Erythrocytic CYB5R enzyme activities were low in all dogs assayed. Owner-reported quality of life ranged from subclinical to occasional exertional syncope. Two previously reported and two novel CYB5R3 missense variants were identified among the methemoglobinemic cohort and were predicted to impair enzyme function. Two variants were recurrent: a homozygous Ile194Leu substitution was found in Pomeranians and other small dogs, and a homozygous Arg219Pro change occurred predominately in pit bull terriers. The other two variants were Thr202Ala and Gly76Ser substitutions in single dogs. Of the two common *CYB5R3* genotypes, Arg219Pro was associated with a more severe metabolic phenotype. We conclude that CYB5R3 deficiency is the predominate cause of canine hereditary methemoglobinemia. Although this finding is unlikely to alter the clinical approach to hereditary methemoglobinemia in dogs, it demonstrates the possibility of how genotype–phenotype cohort analysis might facilitate precision medicine in the future in veterinary medicine.

## Introduction

Iron in hemoglobin (Hb) must be in the ferrous (Fe^+2^) state to bind oxygen. Methemoglobin (MetHb) forms when iron moieties are oxidized to the ferric state (Fe^+3^). It is estimated that 2–3% of Hb is oxidized to MetHb each day in dogs from oxidants generated by normal metabolic reactions and from the spontaneous autoxidation of oxyhemoglobin^1^. Methemoglobin concentration is normally maintained at < 2% of total Hb by virtue of the cytochrome b_5_ reductase (CYB5R)/cytochrome b_5_ redox pathway^1–5^. Methemoglobinemia results when there is a defect in this pathway, or there is exposure to high levels of exogenous oxidants that overwhelm the reductive capacity of this pathway^1–5^. Exogenous oxidants generally cause sufficient damage to induce an accompanying hemolytic anemia^4^.

In contrast, defects in the CYB5R/cytochrome b_5_ redox pathway result in methemoglobinemia without anemia, and some patients even exhibit a mild erythrocytosis^4^. Hereditary methemoglobinemia in dogs can be found incidentally, when cyanotic mucous membranes and brown colored blood are identified on routine physical examinations and during surgical procedures, respectively. However, in some dogs, hereditary methemoglobinemia is associated with clinical signs like decreased energy or exertional syncope, which are consequences of tissue hypoxia^6–10^.

Deficiency of the soluble type I CYB5R (EC 1.6.2.2), a flavoprotein of the transhydrogenase family of oxidoreductase enzymes, in erythrocytes is the most common cause for hereditary methemoglobinemia in humans (OMIM 250800)^5^. The same is purported to be true in dogs (OMIA 002131-9615)^6,11,12^ and cats (OMIA 002131-9685)^13,14^ based upon a few case reports. Other causes of hereditary methemoglobinemia in humans are exceedingly rare and include cytochrome b_5_ deficiency and hemoglobin M disorders, of which only the former has been suspected in a single dog^15–18^. While CYB5R deficiency is decidedly the most common cause of hereditary methemoglobinemia in humans^5^, no large causative survey has been conducted in dogs. Moreover, associations between phenotype (clinical signs and biochemical derangements) and genotype have been established in humans but not yet in dogs^19^.

This prospective study had two objectives: firstly, to characterize the molecular basis and clinical spectrum in a cohort of dogs with hereditary methemoglobinemia; and secondly, to determine if there are biochemical or clinical phenotypic differences ascribed to separate *CYB5R3* gene variants.

## Results

### Animal population

Thirty dogs were enrolled in this study. Dogs were assessed using clinical (quality of life [QOL]; n = 29), metabolic (MetHb concentration, n = 30; Hb concentration, n = 24; CYB5R enzyme activity, n = 27), and genetic *CYB5R3* variant (n = 30) evaluations. There were 13 owner-reported mixed-breed dogs and 17 purebred dogs: pit bull terriers (10), Pomeranians (five), and one each of rat terrier and Chihuahua. There were 18 spayed females, 10 castrated males, and two intact males. The mean age of dogs at the time of inclusion was 4.9 years (SD ± 2.8). Eight dogs had a historical diagnosis of persistent, unexplained cyanosis and methemoglobinemia for a variable period of time before inclusion. Although pedigrees were not available, and no parentage studies were performed, six mixed-breed dogs were presumed to be closely related, because they had a similar small-sized appearance and were housed together when confiscated by animal shelter services.

### Methemoglobin, hemoglobin, and cytochrome b_5_ reductase activity measurements

Dogs with CYB5R deficiency had higher blood Hb concentrations (median, interquartile range [IQR]; 19.3 g/dL, 18.2–20.6; n = 24) than simultaneously tested unaffected control dogs (median, IQR; 15.5 g/dL, 14.4–16.5; n = 22, *P* < 0.001). Likewise, MetHb concentrations were higher in dogs with CYB5R deficiency (median, IQR; 23.4%, 17.0–32.0; n = 30) than control dogs (median, IQR; 2.4%, 2.2–3.1; n = 25, *P* < 0.0001). Median erythrocyte CYB5R enzyme activity in dogs with CYB5R deficiency was 10.9% (IQR, 5.0–24.1; n = 27). The correlation between Hb concentration and both log transformed MetHb (r[20] = 0.18, *P* = 0.40) and log transformed CYB5R enzyme activity (r[21] = − 0.13, *P* = 0.56) was weak and not significant, however, there was a negative correlation between log transformed MetHb and log transformed CYB5R enzyme activity (r[25] = − 0.51, *P* = 0.006).

### Molecular genetic analyses of dogs with methemoglobinemia

The first dog to be included in the current study was the same dog previously analyzed by whole genome sequencing and reported to harbor two heterozygous missense variants in *CYB5R3*: Gly76Ser and Ile194Leu^6^. The previous report utilized the Ensembl annotation (ENSCAFT00000001475.4) to report predicted amino acid substitutions. The National Center for Biotechnology Information (NCBI) canine genome annotation was used in the current study. None of the subsequently studied dogs were found to carry the Gly76Ser variant; however, the Ile194Leu variant was found in the homozygous state in five Pomeranians, one Chihuahua, one rat terrier, and seven mixed-breed dogs of small stature. When a dog’s methemoglobinemia could not be explained by previously identified *CYB5R3* variants, the coding exons of this gene were sequenced. This led to the identification of a homozygous Thr202Ala variant in one mixed-breed dog and a homozygous Arg219Pro variant in seven pit bull terriers and four mixed-breed dogs. In addition, three other pit bull terriers were heterozygous for the Arg219Pro variant, but no other missense variants were found in their exons. The chromosomal positions, cDNA changes, and predicted amino acid substitutions for the four *CYB5R3* variants are presented in Table 1.

**Table 1:** Coordinates and functionality predictions for *CYB5R3* missense variants in dogs with hereditary methemoglobinemia.

Genomic change^a^	cDNA change^b^	Amino acid change^c^	PROVEAN/SIFT analyses^d^
10:22,832,962G > A	c.227G > A	p.Gly76Ser	Deleterious/not tolerated
10:22,836,951A > C	c.580A > C	p.Ile194Leu	Neutral/not tolerated
10:22,841,895G > C	c.656G > C	p.Arg219Pro	Deleterious/not tolerated
10:22,836,975A > G	c.604A > C	p.Thr202Ala	Deleterious/not tolerated

^a^Numbered according to CanFam3.1

^b^Numbered according to NM_001048084.

^c^Numbered according to NP_001041549.

^d^Predictions of the effect of the amino acid substitution [SIFT^41^, PROVEAN (https://provean.jcvi.org)].

### CYB5R3 variant assessment and in-silico protein analyses

**Figure 1:**
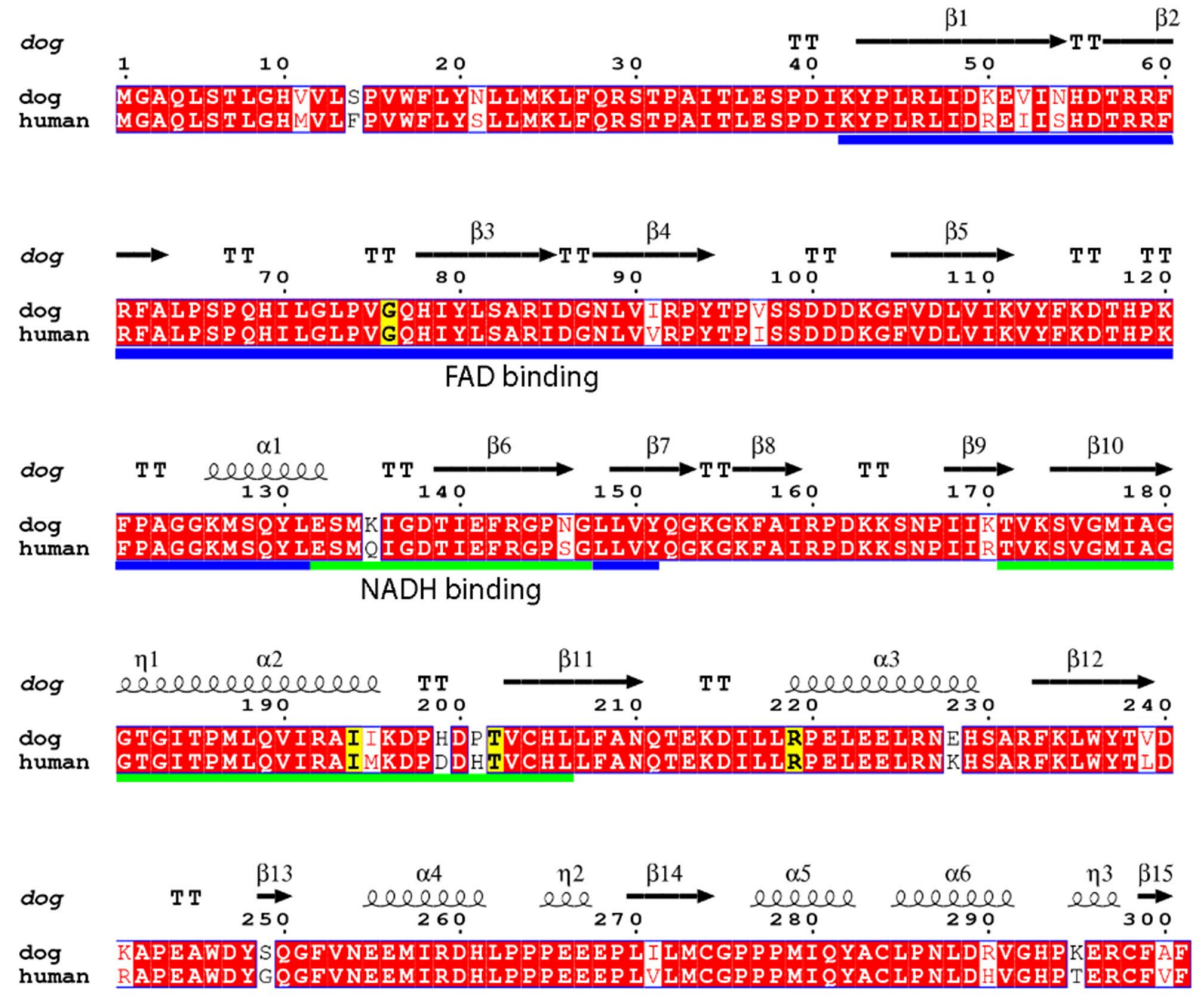
Pair-wise sequence alignment of normal human and canine *CYB5R3* gene regions and sites of methemoglobinemia-associated variants in dogs. The CYB5R amino acid sequences were aligned using the EMBOSS/EMBL-EBI server^37^ and visualized by ENDSCRIPT program^38^. Residue numbers are labeled according to the canine sequence. The completely conserved CYB5R amino acids between dog and human residues are shaded in red and the variants are shaded in yellow. Secondary elements of CYB5R derived from canine crystal structure are drawn above the alignment. The nicotinamide adenine dinucleotide (NADH) (blue) and flavin adenine dinucleotide (FAD) (green) domains are indicated by solid lines under the alignment. UNIPROT database accession numbers are: P00387 (human) and Q0X0E5 (canine).

The canine (NP_001041549.1) and human CYB5R (NP_000389.1) protein sequence is highly conserved (91%) with only 22 of 301 amino acid differences (Fig. 1). Moreover, multiple alignment with 250 UNIPROT^20^ entries for *CYB5R3* (eukaryote, representing 135 species) amino acids Gly76 and Thr202 are completely conserved (100%), Arg219 is conserved in 97% of the sequences, being rarely substituted by His, Gln, or Lys. Ile194 is conserved in 79% of the sequences, being rarely replaced by Val, Met, or Leu. Structurally, amino acids Gly76, Ile194, and Thr202 lie at the end of the FAD/NADH binding region within turn 3, α-helix 2 and β-sheet 11 (Fig. 1 and Supplemental Fig. 1). In contrast, Arg219 is a surface residue located at the beginning of α-helix 3 between β-sheets 11 and 12. The amino acid substitution prediction algorithms Sorting Intolerant From Tolerant (SIFT) and DUET predicted each of the variants to affect protein function (Table 1 and Supplemental Table S1). The Protein Variation Effect Analyzer (PROVEAN) also predicted each of the variants, except Ile194Leu (neutral change), to affect function (Table 1 and Supplemental Table S1).

### Quality of life survey

**Figure 2:**
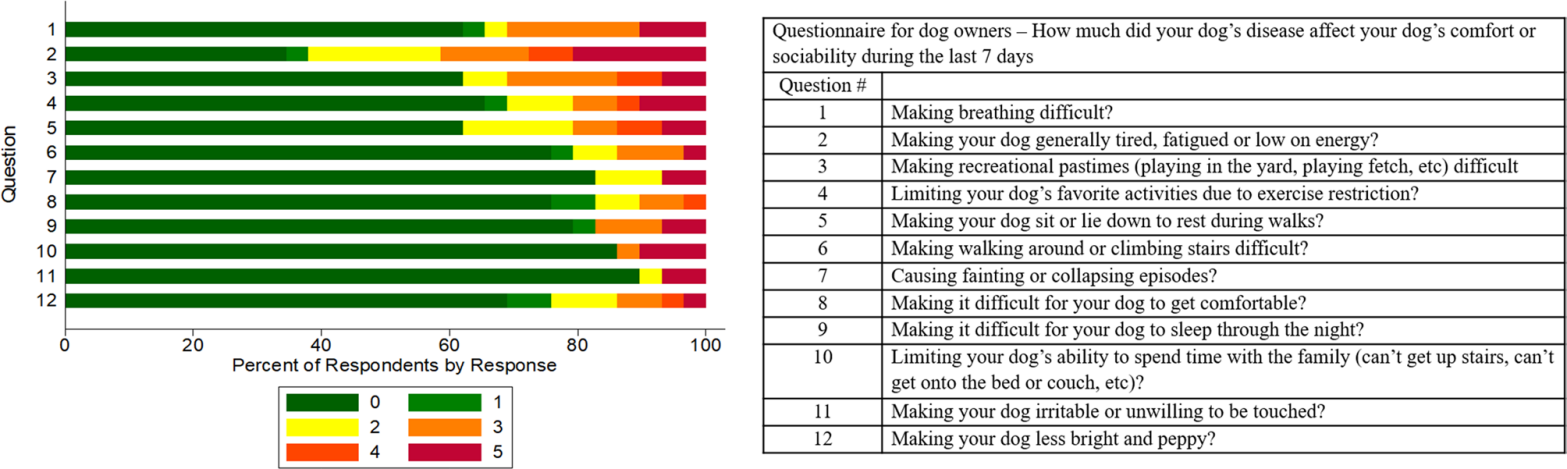
Distribution of the owner responses for each of 12 quality of life (QOL) questions for dogs with hereditary methemoglobinemia. A modified functional evaluation of cardiac health (FETCH)-questionnaire was used with questions that were not pertinent to methemoglobinemia omitted^33^. Color shows percent of response for each response option from 0 (not at all) to 5 (very much) for each question.

Scores for quality of life (QOL) were recorded by dog owners for 29 of the 30 dogs with CYB5R deficiency, with the median overall score (i.e. sum of scores for all 12 questions) of 4 (IQR, 0–13) out of a possible maximal 60 score, where a higher score reflected a worse owner perceived QOL. Twenty (70%) of the dogs had an overall score > 0, 13 (45%) had an overall score of > 5, and 4 (14%) had an overall score of ≥ 30 (Supplemental Fig. 2). Five dogs without recognized comorbid conditions experienced exertional syncopal episodes with median MetHb and Hb concentrations of 26% (IQR, 15.0–32.6; reference < 4%) and 19.3 g/dL (IQR, 18.4–20.7; reference 12–18 g/dL). The distribution of the QOL scores was zero inflated and right skewed (Fig. 2). Chi-squared goodness of fit analysis of the observed counts of dogs with a score > 0 for each question (Supplemental Table S2) showed that observed frequencies differed from expected frequencies according to the likelihood-ratio chi-square test (*P* = 0.048). Analysis of the Pearson residuals generated from the chi-squared goodness of fit test showed that the answer to Question #2, “How much did your dog’s disease affect your dog’s comfort or sociability during the last 7 days” by: “Making your dog generally tired, fatigued or low on energy?”, had a higher number of non-zero responses than expected with a Pearson residual of 3.4, which was greater than the critical value of 2.9. This indicates that more dogs were reported to have at least some indication of tiredness, fatigue, or low-energy than expected as compared to any of the other QOL questions. However, there was no concurrent comparison made to breed- and age-matched unaffected control dogs.

### Phenotype to genotype comparisons in dogs homozygous for Ile194Leu and Arg219Pro variants in CYB5R

**Figure 3:**
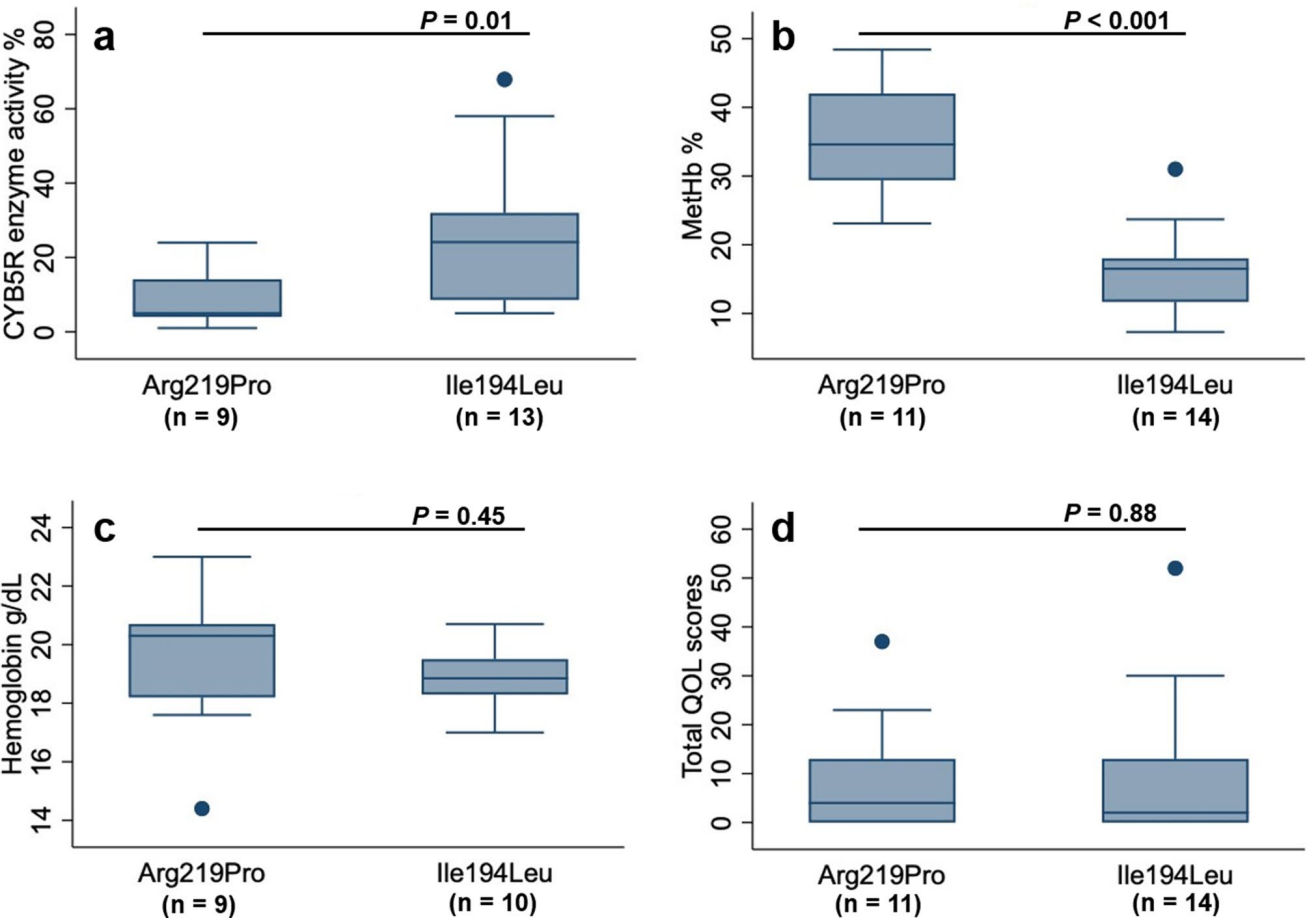
Comparison of hematological, biochemical, and quality of life (QOL) parameters for 24 dogs homozygous for Arg219Pro or the Ile194Leu *CYB5R3* gene variants. Box plots comparing **(a)** erythrocyte cytochrome b_5_ reductase (CYB5R) enzyme activity, **(b)** methemoglobin (MetHb) concentrations, **(c)** hemoglobin concentrations and, **(d)** overall total QOL scores (i.e. sum of total scores for all 12 questions). The horizontal line is at the median, the boxes span from the 25th to 75th percentile, and whiskers extend from each quartile to minimum or maximum. The closed circles represent data points beyond 1.5 × interquartile range.

Initial comparisons included all dogs that were homozygous for either the Arg219Pro or Ile194Leu variants. Differences were identified between the two *CYB5R3* variants regarding MetHb concentration and erythrocyte CYB5R enzyme activity (Fig. 3 and Table 2). There was no difference found for clinical QOL scores between variants either for the total sum (*P* = 0.88, Fig. 3), for the count of dogs with total scores > 5 (*P* = 0.53), or for the sum of each individual question (all *P* > 0.05 even without correction for multiple comparisons). Exertional syncopal episodes were reported in dogs with Arg219Pro or with Ile194Leu variants.

**Table 2:** Comparative laboratory test results and total quality of life scores for dogs with hereditary methemoglobinemia associated with homozygous Arg219Pro or Ile194Leu missense *CYB5R3* gene variants. Purebred comparisons can be found in Supplemental Table S4.

Variable	Arg219Pro	Ile194Leu	*P*-value^c^
Number of dogs	11	14	
Hb concentration (g/dL)^a,^* (normal 12–18 g/dL)	19.6 (2.5)^d^	18.9 (1.2)^d^	0.45
Methemoglobin concentration (%)^b,^* (normal ≤ 4%)	34.6 (29.4–42.0)	16.5 (11.7–18.0)	**0.001**
Erythrocytic CYB5R enzyme activity (%)^b^ (normal 100 + 25%)	5.0 (4.0–14.1)^e^	24.1 (8.6–32.0)^e^	**0.01**
QOL score sum (0–60)^b^ (no signs = 0, maximum = 60)	4.0 (0–13.0)	2.0 (0–13.0)	0.88
Number of dogs with QOL score sum of > 5, n (%)	7 (64)	8 (57)	0.53
Number of dogs with exertional syncope, n (%)	2 (18)	2 (14)	–-

*Hb* hemoglobin, *CYB5R* cytochrome b_5_ reductase, *n* number, *QOL* quality of life.

*Significant difference of both variants to simultaneously sent unaffected controls.

^a^Data presented as mean (standard deviation).

^b^Data presented as median (interquartile range).

^c^Significant *P*-values bolded.

^d^Hemoglobin concentration available in Arg219Pro homozygous dogs (n = 9) and Ile194Leu homozygous dogs (n = 10).

^e^Erythrocytic CYB5R enzyme activity available in Arg219Pro (n = 9) and Ile194Leu (n = 13).

Next, metabolic parameters and QOL scores were compared between pit bull terriers homozygous for the Arg219Pro variant and Pomeranians that were Ile194Leu homozygotes. Pit bull terriers had higher MetHb concentration than Pomeranians (*P* = 0.01). There was no difference found for total QOL scores (*P* = 0.43), Hb concentrations (*P* = 0.18), or erythrocyte CYB5R enzyme activities (*P* = 0.61; Supplemental Table S3).

Laboratory test results and total QOL scores for dogs heterozygous for the Arg219Pro variant as well as the two individual dogs that were either homozygous for the Thr201Ala variant or heterozygous for Gly76Ser/Ile194Leu variants can be found in Supplemental Table S4.

## Discussion

Here we characterize a cohort of 30 dogs with persistent methemoglobinemia with respect to clinical spectrum, biochemical derangements, and molecular defects as well as provide phenotype-genotype correlations for two common breed-related *CYB5R3* gene variants. The Arg219Pro missense variant seen in pit bull terriers and mixed-breed dogs was associated with similarly low QOL scores, but higher MetHb concentrations, and lower CYB5R enzyme activities compared to the Ile194Leu substitution seen in Pomeranians and at least two other small dog breeds.

Two novel missense variants in addition to two previously reported variants in the *CYB5R3* gene were identified among 30 dogs with persistent methemoglobinemia due to CYB5R deficiency^6,11,12^. Two variants were clearly breed-associated: pit bull terriers carried at least one copy of the Arg219Pro variant, while all Pomeranians, as previously reported^11,12^, and other breeds were homozygous for the Ile194Leu variant. A survey was not performed to assess the prevalence of these variants in these breeds. The other two variants were each only seen in a single mixed-breed dog and thus may be rare or private variants.

Hereditary methemoglobinemia due to CYB5R deficiency is an autosomal recessive trait in all species studied^21,22^. Thus, we expected to find biallelic variants in all of the affected dogs. Nonetheless, three affected pit bull terriers and one affected mixed-breed dog were heterozygous for the Arg219Pro variant, and no other mutant allele was found. Because we only sequenced the coding exons and adjacent intronic regions, but not the deep intronic and regulatory regions, we may have missed pathogenic variants on the trans chromosome.

Based upon on-line predictive algorithms (DUET, PROVEAN, SIFT) all four variants were estimated to affect enzyme function and/or stability (with one exception, the PROVEAN model rated the Ile194Leu as a neutral change). The erythrocytic CYB5R enzyme activity measurements from dogs with each of the four respective variants substantiates the results from these predictive algorithms and are in line with previous reporting for the Ile194Leu variant^11^. Two of the four *CYB5R3* variants observed in this study have corollaries in humans with CYB5R deficiencies. The Gly76Ser amino acid substitution, identified in one dog has previously been reported to cause CYB5R deficiency in humans^23^. Furthermore, the Arg219Pro variant is in an adjacent position to the Lys218Pro variant, which is associated with CYB5R deficiency in humans^24,25^. Presumably, the proline substitution at position 218 or 219 in the enzyme exerts the same negative effect on protein function. The Ile194Leu and Thr202Ala substitutions have not been observed previously in humans with CYB5R deficiency; however, nearby amino acid substitutions (Arg192Cys and Cys204Arg) are associated with methemoglobinemia in humans^21,26^.

The erythrocyte CYB5R enzyme activities were significantly lower in dogs homozygous for Arg219Pro versus the Ile194Leu variant, which likely led to the higher MetHb concentrations in Arg219Pro homozygotes. Humans, dogs, and cats with hereditary methemoglobinemia develop a compensatory erythrocytosis (polycythemia), which was also seen in this cohort of dogs with erythrocytic CYB5R deficiency, when compared to unaffected control dogs^21,27^. However, there was no significant difference between the Arg219Pro homozygotes and the Ile194Leu homozygotes. It should be noted that at the time of enrollment, the dogs with the Arg219Pro variant were about half the age as those with the Ile194Leu variant. No studies were pursued to follow these cases and determine whether the erythrocytosis in the dogs with the Arg219Pro variant became more severe over time.

Persistent cyanosis was the most consistent clinical finding in all dogs and the main reason for further clinical evaluation. However, these dogs may have had other unrecognized clinical signs; detailed medical records from primary care veterinarians were not available for review. Based upon owner completed QOL questionnaires, clinical signs were variable and generally mild including decreased activity and exercise tolerance, and occasionally exertional syncope. These clinical manifestations are similar to previous case reports in dogs^6–10^ as well as humans with CYB5R deficiency^19,22,25,27–32^.

A QOL questionnaire modified from the published functional evaluation of cardiac health (FETCH) tool used to assess dogs with cardiopulmonary diseases was used in this study^33^. The median overall QOL scores were low, indicating that dog owners perceived their dog to be minimally affected, and there were no significant differences between those dogs homozygous with the Arg219Pro variant versus the Ile194Leu homozygotes. Again, a difference may have been missed, because the dogs were only evaluated by the owner on a single occasion and not at the same age. Furthermore, the number of dogs in each group was small (type II error) and owners’ inexperience may have limited the recognition of clinical abnormalities. Similarly, humans with hereditary methemoglobinemia adapt well to their condition due to the chronicity as well as slow or absent progression by reducing strenuous exercise and the development of a secondary polycythemia. Furthermore, the lack of correlation between clinical signs and magnitude of methemoglobinemia in this study is corroborated with reports in humans with CYB5R deficiency^28,31^.

There is very limited information regarding genotype–phenotype relationships in humans with CYB5R deficiency, likely because of the rarity of the disease, mild overall clinical symptoms, as well as vast heterogeneity of genotypes. When comparing the phenotype to genotype based on CYB5R enzyme regions affected, the variants involving the linker domain yielded less severe clinical phenotypes in humans than those affecting the FAD-binding lobe and NADH-binding domain, where the *CYB5R3* variants in our study were found^19^. However, it should again be emphasized that hereditary methemoglobinemia is an illness to which humans and dogs can adapt well with appropriate lifestyle or husbandry adjustments and often only require treatment to avoid or manage any crises related to severe tissue hypoxia, preparation for anesthetic procedures, or with the presence of comorbid diseases that decrease arterial oxygen tension or oxygen carrying capacity. All 30 affected dogs showed erythrocytic CYB5R deficiency and presumably none had any of the other causes of hereditary methemoglobinemia, which have only rarely been described in humans^16–18^. Indeed, CYB5R deficiency is the main reason for hereditary methemoglobinemia in humans and results from our study suggest the same is true in dogs^1,5^.

In conclusion, all dogs with methemoglobinemia in this 30-dog cohort study had CYB5R deficiency. The clinical signs varied in type and severity, but were generally mild and well-tolerated. Of the two most common *CYB5R3* variants, the Arg219Pro variant in pit bull terriers appeared to cause a more severe metabolic phenotype than the Ile194Leu variant in Pomeranians and other breeds. The four *CYB5R3* variants identified can be readily used as genetic screening tests in dogs to diagnose hereditary methemoglobinemia.

## Materials and methods

### Dogs, blood samples, and criteria for selection of cases

Board-certified small animal internists, cardiologists, and anesthesiologists were contacted directly by one author (JAJ) or via postings on each colleges’ respective online list-serve used for communication among veterinary specialists for the recruitment of eligible dogs. One previously reported dog with CYB5R deficiency was included^6^. Dogs with unexplained persistent mild to severe cyanosis without evidence of cardiopulmonary disease between 2017 and 2019 were eligible for inclusion. The breed, sex, and age were obtained from sample submission forms for the measurements of blood MetHb and Hb concentration, as well as CYB5R enzyme activity testing. Simultaneously collected blood samples from adult healthy control dogs of varied genetic background were sent along with the affected dog sample from the same clinic for direct test result comparison. Written informed consent was obtained from all dog owners along with the completed QOL questionnaire. All animal experiments were performed in accordance with the relevant guidelines and regulations set forth by the University of Missouri Animal Care and Use Committee represented by the approved study protocol # 9154. The University of Missouri Animal Care and Use Committee has waived the need for ethical approval and guidelines for conducting questionnaire with dog owners in this study.

Fresh ethylenediaminetetraacetic acid-anticoagulated venous blood (2–4 ml) samples were sent for Hb and MetHb quantification, and CYB5R enzyme activity determination with ice packs overnight to the PennGen Laboratories of the School of Veterinary Medicine at the University of Pennsylvania. Blood samples from four dogs were instead sent for MetHb determination to the Veterinary Diagnostic Laboratory at the University of Wisconsin. Left-over samples were kept refrigerated and after testing sent to the University of Missouri Veterinary College of Veterinary Medicine for molecular genetic studies.

### Methemoglobin, hemoglobin, and cytochrome b_5_ reductase enzyme activity measurements

Erythrocytic MetHb and Hb concentrations as well as CYB5R enzyme activities were quantified with previously established standard procedures using spectrophotometric assays^34,35^. Methemoglobin concentrations were reported as the percentage of total Hb concentration. Cytochrome b_5_ reductase enzyme activity was reported as percentage of a concurrently measured shipped sample from a healthy unaffected control dog designated to have 100% CYB5R enzyme activity.

### Quality of life questionnaire and survey

Quality of life was assessed by each dog owner with a modified FETCH-questionnaire^33^. Six questions that appeared irrelevant to methemoglobinemia were omitted. There were 12 questions with five possible scores for each question ranging from 0 (not at all affected) to 5 (very much affected) (Fig. 2). Responses for each of the questions were summed to obtain an overall total score for each dog, with possible total scores ranging from 0 to 60.

### Sequencing CYB5R3 exons

Genomic DNA was isolated from anticoagulated blood samples as previously described^36^. Sanger sequencing was used to examine amplicons produced with PCR primers designed from intronic sequences flanking coding exons 2 through 8 of canine *CYB5R3.* The primer sequences are shown in Supplement Table S5. Amplification reactions were done with GoTaq Flexi DNA Polymerase Kit (Promega, Madison, WI). The initial 95 °C denaturation was for two min, followed by 40 cycles of denaturation at 95 °C for 15 s, primer annealing at 60 °C for 30 s, extension at 72 °C for 30 s, and a final extension at 72 °C for two min. The resulting amplicons were purified with QIAquick spin columns (QIAGEN Germantown, MD) and submitted for bidirectional Sanger sequencing to Sequetech Corporation (Mountain View, CA).

### Genotyping for CYB5R3 missense variants

Assays were devised to genotype individual canine DNA samples for four *CYB5R3* missense variants. A PCR-restriction fragment length polymorphism assay with restriction enzyme *TspRI* was used for a Gly76Ser variant. For this assay, the PCR primers and amplification conditions were the same as those used for Sanger sequencing of *CYB5R3* exon 3. Allelic discrimination assays were designed for the other three *CYB5R3* missense variants. The oligonucleotide sequences for the allelic discrimination assays are provided in Supplement Table S6.

### In-silico protein analysis of canine CYB5R3

A comparative analysis was conducted between canine and human CYB5R3 protein sequences using EMBOSS needle^37^, BIOVIA Discovery Studio Visualizer^38^, DUET^39,40^, PROVEAN (Dessault Systems, Waltham, MA, USA), SIFT^41^, and Human Gene Mutation Database (HGMD)^42^ to assess linear and in silico three-dimensional sequence alignment, protein stability, and comparison to likely pathogenic variants in humans.

### Statistical analysis

All statistical tests were carried out using commercial statistical software programs (Stata Statistical Software, StatCorp LLC, College Station, TX). Normality of all variables was assessed using visual inspection of the histograms and a Shapiro–Wilk test. A dog was considered to have CYB5R deficiency based on two criteria: first, erythrocytic CYB5R enzyme activity was < 30% than a simultaneously measured unaffected control dog, and second, a disease-associated variant in the *CYB5R3* gene was identified. For linear analyses, non-normally distributed variables were log transformed (MetHb concentration and erythrocytic CYB5R enzyme activity). The Kolmogorov–Smirnov test for equality of distribution was used to compare the distributions of age, MetHb and Hb concentrations. For all tests excluding analysis of the chi-square residuals for the individual QOL questions, a *P*-value of < 0.05 was considered significant.

The MetHb and Hb concentrations were compared between all dogs with CYB5R deficiency and controls using a Wilcoxon rank-sum test. The correlation between Hb and log transformed MetHb concentration as well as log transformed MetHb concentration and log transformed CYB5R enzyme activity were compared using Pearson’s correlation. Metabolic values and QOL survey data were compared for the homozygotes with the two most common missense variants, Ile194Leu and Arg219Pro, as well as for pit bull terriers with the Arg219Pro homozygous variant and Pomeranians with the Ile194Leu homozygous variant. The total score from the QOL survey was evaluated as a continuous variable using a Wilcoxon rank-sum test as well as a binary variable based upon the data distribution clinical (score > 5)/subclinical (score ≤ 5) using Fisher’s exact.

The observed and expected frequencies for non-zero scores were compared for individual survey items using a likelihood-ratio chi-square test to determine whether responses differed by question. Pearson residuals from the chi-square test were analyzed to determine which question’s observed count differed from expected by calculating a Bonferroni adjustment to the z critical value of 1.96 to compensate for the large number of cells in the contingency table (24 cells) to arrive at an α of 0.002, resulting in a critical value of + /− 2.9.

## Supplementary Information

Supplementary Information.

## Data Availability

Data to be made available upon request to the corresponding author.

## Abbreviations

CYB5R: Cytochrome b_5_ reductase
FETCH: Functional evaluation of cardiac health
Hb: Hemoglobin
IQR: Interquartile range
MetHb: Methemoglobin
NCBI: National Center for Biotechnology Information
PROVEAN: Protein variation effect analyzer
SIFT: Sorting intolerant from tolerant
QOL: Quality of life
