# Supplementary material for: Clinical, metabolic, and molecular genetic characterization of hereditary methemoglobinemia caused by cytochrome b5 reductase deficiency in 30 dogs
Source: Sci Rep. 2020 Dec 8;10:21399. doi: 10.1038/s41598-020-78391-2 (PMC7723051; doi:10.1038/s41598-020-78391-2)

**Supplemental tables and figures**

Clinical, metabolic, and molecular genetic characterization of hereditary methemoglobinemia caused by cytochrome b_5_ reductase deficiency in 30 dogs

J.A. Jaffey,^a,b*^ N.S. Reading,^c^ O. Abdulmalik,^d^ R. Kreisler,^e^ G. Bullock,^f^ A. Wiest,^g^ N.A. Villani,^f^ T. Mhlanga-Mutangadura,^f^ G.S. Johnson,^f^ L.A. Cohn,^b^ N. Isaza,^h^ J.W. Harvey,^i^ U. Giger^g^

**Supplemental Table S1.** Computational scores for functional and free energy changes due to amino acid substitutions in cytochrome b_5_ reductase of affected dogs. PROVEAN analysis provides sequence analysis to predict the effect of variants on protein function (neutral threshold > -2.5). Values below the threshold denotes a predicted deleterious effect on protein function. DUET analysis uses changes in the folding free energy (ΔΔG) to predict the effect sequence variation has on protein stability. Negative values correspond to variants with destabilizing effects and positive values correspond to variants with stabilizing effects compared to wild-type.

| ***CYB5R3* gene variant** | **PROVEAN**  **(score)** | **DUET**  **(ΔΔG)** |
| --- | --- | --- |
| Gly76Ser | -5.868 | -2.285 |
| Ile194Leu | -1.877 | -0.819 |
| Thr202Ala | -4.780 | -0.665 |
| Arg219Pro | -6.726 | -1.097 |

**Supplemental Table S2.** Comparison of observed and expected number of clinical responses for each quality of life survey question in 29 dogs with persistent methemoglobinemia due to cytochrome b_5_ reductase deficiency.

| **Question*** | **Observed** | **Expected** | **Observed - Expected** | **Pearson Residuals** |
| --- | --- | --- | --- | --- |
| 1 | 11 | 9.5 | 1.5 | 0.487 |
| 2 | 20 | 9.5 | 10.5 | 3.407** |
| 3 | 12 | 9.5 | 2.5 | 0.811 |
| 4 | 11 | 9.5 | 1.5 | 0.487 |
| 5 | 12 | 9.5 | 2.5 | 0.811 |
| 6 | 8 | 9.5 | -1.5 | -0.487 |
| 7 | 6 | 9.5 | -3.5 | -1.136 |
| 8 | 8 | 9.5 | -1.5 | -0.487 |
| 9 | 7 | 9.5 | -2.5 | -0.811 |
| 10 | 5 | 9.5 | -4.5 | -1.460 |
| 11 | 4 | 9.5 | -5.5 | -1.784 |
| 12 | 10 | 9.5 | 0.5 | 0.162 |

* Quality of life was assessed by each dog owner on a single occasion at the time of enrollment with a modified FETCH-questionnaire.

** The Pearson residuals for Question #2 are higher than the Bonferroni adjusted critical value of +/- 2.9 indicating that there were more non-zero answers than expected.

**Supplemental Table S3:** Comparative laboratory test results and total quality of life scores for pit bull terriers with Arg219Pro homozygous variant and Pomeranians with Ile194Leu homozygous variant in the *CYB5R3* gene.

| **Variable** | **Pit bull terriers**  **Arg219Pro**  **Homozygous** | **Pomeranians**  **Ile194Leu**  **Homozygous** | ***P*-value** |
| --- | --- | --- | --- |
| Number of dogs | 7 | 5 |  |
| Hb concentration (g/dL)  [Normal 12-18 g/dL] | 20.3 [18.2-20.7] | 18.3 [17.3-18.4] | 0.18 |
| Methemoglobin concentration (%)  [Normal ≤ 4%] | 34.6 [26.0-45.3] | 18.0 [11.4-24.5] | **0.01** |
| Erythrocytic CYB5R enzyme activity (%) | 6.2 [2.1-23.5] | 6.4 [5.2-21.6] | 0.61 |
| QOL score sum (0-60) | 13.0 [3.0-23.0] | 10.0 [1.0-16.5] | 0.43 |

Hb, hemoglobin; CYB5R, cytochrome b_5_ reductase; n, number; QOL, quality of life

Data presented as median [interquartile range]

Significant *P*-values bolded

**Supplemental Table S4.** Laboratory test results and total quality of life scores for dogs with hereditary methemoglobinemia associated with Arg219Pro (heterozygotes), Thr201Ala (homozygote), and Gly76Ser/Ile194Leu (heterozygote) missense *CYB5R3* gene variants.

| **Variable** | **Arg219Pro Heterozygotes** | **Thr201Ala Homozygote** | **Gly76Ser/Ile194Leu Heterozygote** |
| --- | --- | --- | --- |
| Number of dogs | 3 | 1 | 1 |
| Hb concentration (g/dL)^a^  [Normal 12-18 g/dL] | 19.4 [19.3-21.4] | 16.4 | 18.6 |
| Methemoglobin concentration (%)^a^  [Normal ≤ 4%] | 30.8 [30.6-33.7] | 21.3 | 20.0 |
| Erythrocytic CYB5R enzyme activity (%)^a^  [Normal 100 + 25%] | 6.0 [1.0-10.9] | 14.0 | 5.8 |
| Number of dogs with QOL score sum (0-60)^a^  [No signs = 0, maximum = 60] | 21.5 [8.0-35.0]^b^ | 4.0 | 38.0 |

Hb, hemoglobin; CYB5R, cytochrome b_5_ reductase; QOL, quality of life

^a^Data presented as median [interquartile range]

^b^Quality of life score not available for one dog

**Supplemental Table S5:** PCR primer sequences for amplification of canine *CYB5R3* exons and adjacent intronic regions.

| **Exon**  **Number** | **Forward/Reverse Primer Sequences** | **Amplicon**  **Size (bp)** |
| --- | --- | --- |
| 2 | CTCATGGGTCTGTCCTTGTCA/TGCCTTGAGCACACAGTAGGTGA | 270 |
| 3 | ACATGTGGCATCAGTACCCCTT/AGCTCCTACCTCTGACCTAGCAC | 239 |
| 4 | ACCCTGCAGAGATAGTCGGT/ACTCATTCAGAAACCAGGTGCAA | 273 |
| 5 | AGCTCACCCCTATGACCCTG/GGCCTTAGGACATTCCAACCC | 214 |
| 6 | GATGGCCGCCCACCCCTCA/ACCTGACCGTAACCCATGACA | 264 |
| 7 | TCCACGTCACCCCATGTCTGC/CACATTTCAGCCATGCAACCAC | 284 |
| 8 | GCAGCCTGTCCTGACCTCT/AGCACCCTCAGAGACCATGCAG | 220 |
| 9 | AGCTGTACTTGGACTCACCT/ATGTTAAAGAGCAGTGGTAGGGA | 243 |

**Supplemental Table S6:** Oligonucleotide sequences for allelic discrimination assays.

| **Amino Acid Substitution** | **Forward/Reverse Primer DNA Sequence** | **Competing Probe Sequences**  **(Reference Allele/Variant Allele)** |
| --- | --- | --- |
| Arg219Pro | TCCCCAGACTGAGAAGGACATC/ GCTTGAAGCGAGCAGAATGTTC | VIC-CTGCTGCGGCCCGA-MBG/ FAM-CTGCTGCCGCCCGA-MBG |
| Thr202Ala | TGATCCGTGCCATCATCAAAGAC/ ACTGACCTGGTTGGCAAATAGTAG | VIC-ACGACCCCACCGTGTG-NFQ/ FAM-CGACCCCGCCGTGTG-NFQ |
| Ile194Leu | TGCTGTTTTGCAGGCATCAC/ ACTGACCTGGTTGGCAAATAGTAG | VIC-CCGTGCCATCATCA-NFQ/  FAM-TCCGTGCCCTCATCA-NFQ |

**Supplemental Fig. 1.** Three-dimensional visualization model of variant locations associated with canine cytochrome b_5_ reductase (CYB5R) deficiency. The ribbon diagram displays the position of Gly76, Ile194, Thr202, and Arg219 amino acids and enzyme cofactor FAD stylized in a stick representation within the protein. Protein variant locations are in yellow, turn regions are represented in green, alpha helixes in red, beta sheet in blue and random coil in grey. The effects of these amino acid substitutions on protein structure are not shown. The image was developed by modelling canine CYB5R amino acid sequence against the homologous human (Protein data bank ID: 1UMK) CYB5R crystal structures using BIOVIA Discovery Studio Visualizer [38].


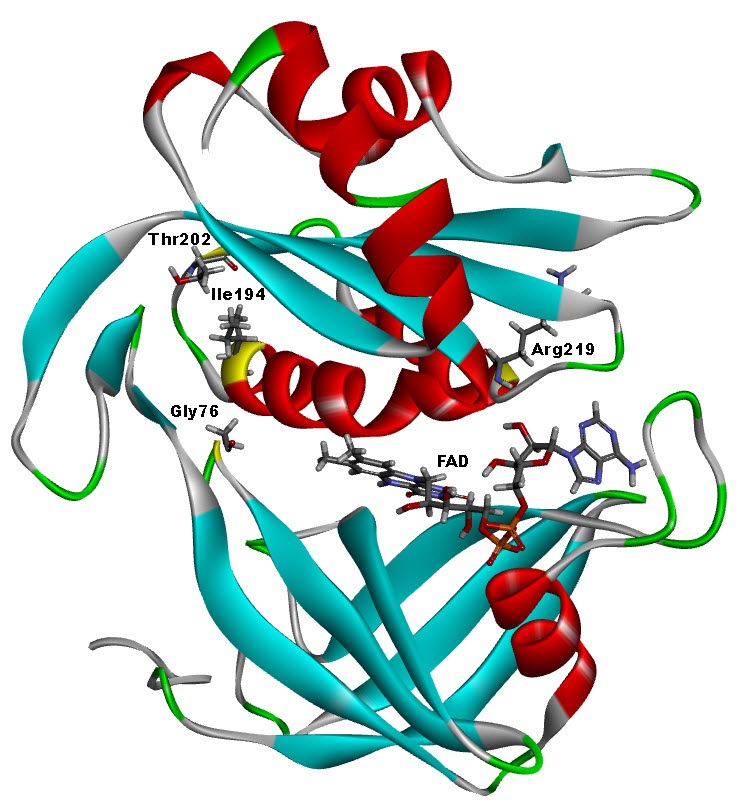


**Supplemental Fig. 2.** Distribution of total score from 12 questions measuring quality of life in 29 dogs with hereditary methemoglobinemia. A modified functional evaluation of cardiac health (FETCH) questionnaire was used to assess quality of life [33].


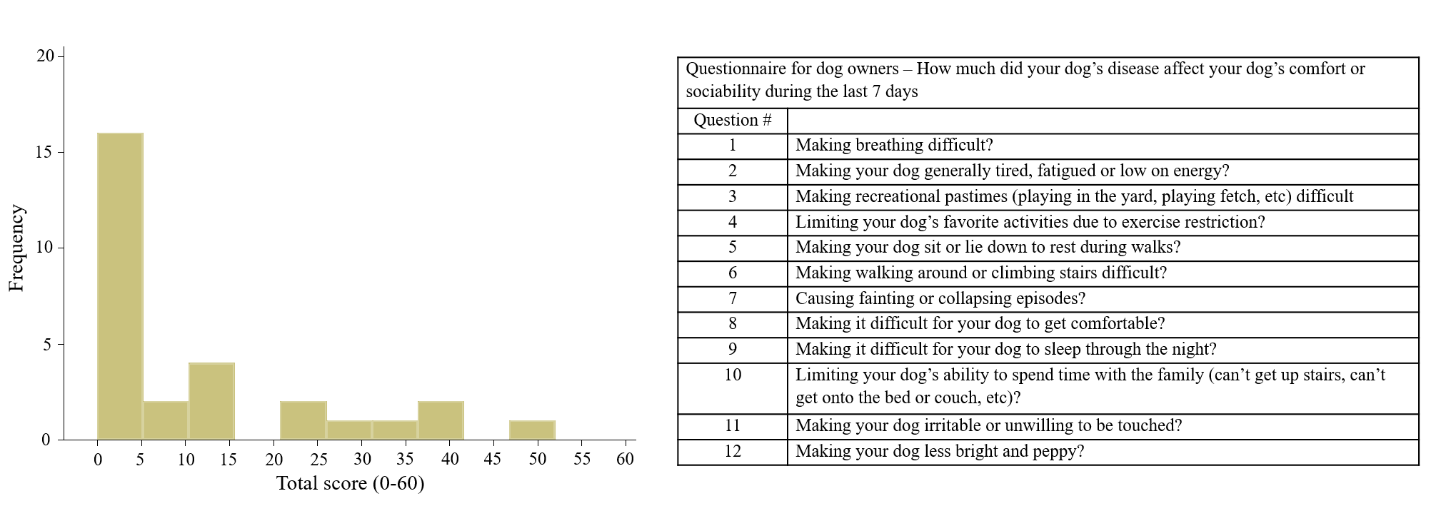

Supplement: Supplementary file 1 — Supplementary Information. [file 41598_2020_78391_MOESM1_ESM.docx]
